# Feasibility of conducting a randomized controlled trial to explore the muscular influence of post‐operative intravenous iron treatment for anaemia after major abdominal surgery

**DOI:** 10.1111/vox.70037

**Published:** 2025-04-22

**Authors:** Beth MacLean, Robert U. Newton, Jayne Lim, Toby Richards

**Affiliations:** ^1^ School of Medicine The University of Western Australia Perth Australia; ^2^ Exercise Medicine Research Institute Edith Cowan University Perth Australia; ^3^ School of Human Movement and Nutrition Sciences University of Queensland Brisbane Australia; ^4^ School of Health, Sport and Bioscience University of East London London UK

**Keywords:** anaemia, clinical trial, haemoglobin measurement, iron deficiency, patient blood management, sarcopenic

## Abstract

**Background and Objectives:**

Iron deficiency anaemia is common in patients recovering from major surgery and is associated with poorer post‐operative outcomes. We designed a randomized controlled trial treating post‐operative anaemia with iron therapy to observe the influence on post‐operative recovery.

**Materials and Methods:**

Anaemic patients (haemoglobin [Hb] < 120 g/L for women, Hb < 130 g/L for men) recovering from major abdominal surgery at Fiona Stanley Hospital were recruited. Patients were double‐blind randomized 1:1 to ferric carboxymaltose (FCM) or saline administered 4 weeks post‐discharge and included in a 12‐week exercise programme. Iron indices, quality of life (QoL) questionnaires and muscle function tests were conducted at 4 weeks (baseline), 8‐, 12‐ and 16‐weeks post‐discharge. This pilot study primarily aimed to assess the feasibility of recruiting 20 patients per intervention arm. Trial registration: ACTRN12622001447741.

**Results:**

Of 205 eligible patients screened between 5 May 2023 and 31 August 2023, only four patients were recruited, of which one completed the study. After randomization to FCM, results obtained from the sole participant that completed the trial suggested a trend towards improvement in QoL outcomes, Hb and muscle function.

**Conclusion:**

Recruitment to a randomized controlled trial exploring the influence of iron therapy on muscle function after major abdominal surgery was not feasible.

**Highlights**

- Anaemia is common in patients recovering from major abdominal surgery.
- Recruiting patients recovering from major abdominal surgery to a randomized controlled trial exploring the impact of intravenous iron on functional recovery was not feasible.
- Key limitations to recruitment included patients not meeting haematological eligibility criteria, lack of interest in the study and transport issues.

## INTRODUCTION

Prior to major surgery, anaemia affects one in three patients and has been associated with poorer post‐operative outcomes, including increased morbidity and mortality, increased length of hospital stay and increased risk of blood transfusion [1]. After major surgery, the incidence of anaemia increases to three in four patients [2].

The most common cause of anaemia is iron deficiency; therefore, iron therapy has been substantially trialled as a treatment for anaemia in the pre‐operative period [1]. However, pre‐operative iron therapy can only be suggested to benefit pre‐ and post‐operative haemoglobin (Hb) concentration and is yet to otherwise conclusively show a direct patient benefit [1].

After major surgery, patient recovery is typically reported in accordance with Enhanced Recovery After Surgery (ERAS), focusing on mobilization, nutrition and discharge [3]. Bedbound critically ill patients admitted to the intensive care unit (ICU) can experience a total skeletal muscle mass loss between 10% and 26% in just 10 days of admission [4]. When muscle mass and strength fall below certain thresholds, as defined by the European Working Group on Sarcopenia in Older People (EWGSOP), a person becomes sarcopenic. Sarcopenia is associated with an increased risk of all‐cause mortality, hospitalization, increased length of hospital stay and threefold increased risk of both developing a physical disability and the occurrence of falling events [5]. Key risk factors for losing muscle mass include periods of inactivity, malnourishment, inflammation and older age (>50 years), which are all factors that frequently present in patients undergoing major surgery [6, 7].

Iron is essential for mitochondrial function, which consequently regulates muscle contraction, oxygen transport and substrate oxidation–reduction reactions [8]. Skeletal muscle tissue contains between 10% and 15% of bodily iron [9]. Therefore, we designed a randomized controlled trial to explore whether using iron therapy to treat post‐operative anaemia could improve muscle function outcomes.

## MATERIALS AND METHODS

### Study design, participants and recruitment

The aim of this trial was to explore the feasibility of recruiting patients who are recovering from major abdominal surgery to a randomized controlled trial exploring the impact of post‐operative intravenous iron on muscle function. Participants recovering from major abdominal surgery at Fiona Stanley Hospital, Western Australia, were approached for recruitment in the abdominal ward within the acute recovery period. Patients were eligible for recruitment if they had undergone a major laparoscopic or open procedure (including organ resection, reconstruction or major adhesiolysis); were anaemic (Hb < 120 g/L for women and <130 g/L for men); were non‐pregnant adults (>18 years old); had not received intravenous iron within the past 3 months; and had no known history of haemochromatosis or known alternate cause for anaemia (i.e., B12 deficiency, folate deficiency or myelodysplasia). The trial was registered as ACTRN12622001447741. Further details regarding the study protocol can be found in the Supporting Information.

### Randomization and masking

Computer generated 1:1 randomization was conducted in blocks of four by an unblinded team member who remained uninvolved in data collection, analysis and patient care. Sealed envelopes were used to deliver randomization to nurses in an outpatient clinic, who were responsible for preparing and delivering the assigned intervention and remained uninvolved in patient care, data collection and analysis. The intervention was concealed from the patient, caregivers, and study members by delivering the preparation through amber tubing and bags.

### Procedures

Patients were randomized 1:1 to receive either 1000 mg of ferric carboxymaltose (FCM), delivered in 250 mL of normal saline (NaCl) or 250 mL of saline (placebo) at an outpatient clinic 4 weeks after discharge from the index surgery. Thereafter, patients were included in a personalized exercise programme guided by an exercise physiologist for a duration of 12 weeks, with 2 × 1‐h sessions delivered per week.

### Outcomes

To address the primary aim of feasibility, a goal of recruiting 40 patients undergoing major surgery, 20 in each intervention arm, was set. To monitor the feasibility of recruitment, a detailed screening log was captured, including the type of surgery performed, Hb, patient eligibility status, acceptability of trial participation and reasons for non‐participation. Descriptive analysis of the data was performed to provide a brief snapshot of the feasibility of recruitment.

Secondary outcomes were recorded at 4 (baseline), 8, 12 and 16 weeks after discharge. These included haematological response (Hb, ferritin, transferrin saturation [TSAT], C‐reactive protein), quality of life (QoL) assessments (SF‐36v2 and EQ‐5D‐5L) and muscle function (handgrip strength, 6‐min walk test, 30‐s sit‐to‐stand test and timed up and go [TUG] test).

### Statistical analysis

Feasibility outcomes are reported descriptively as the number of responders. In terms of secondary outcomes, continuous variables were assessed for normality using Shapiro–Wilk normality testing. Normally distributed data were reported as mean and standard deviation and analysed by *t* test, while abnormally distributed data were reported as median and interquartile range and analysed by Mann–Whitney *U* testing.

## RESULTS

### Recruitment feasibility

**FIGURE 1:**
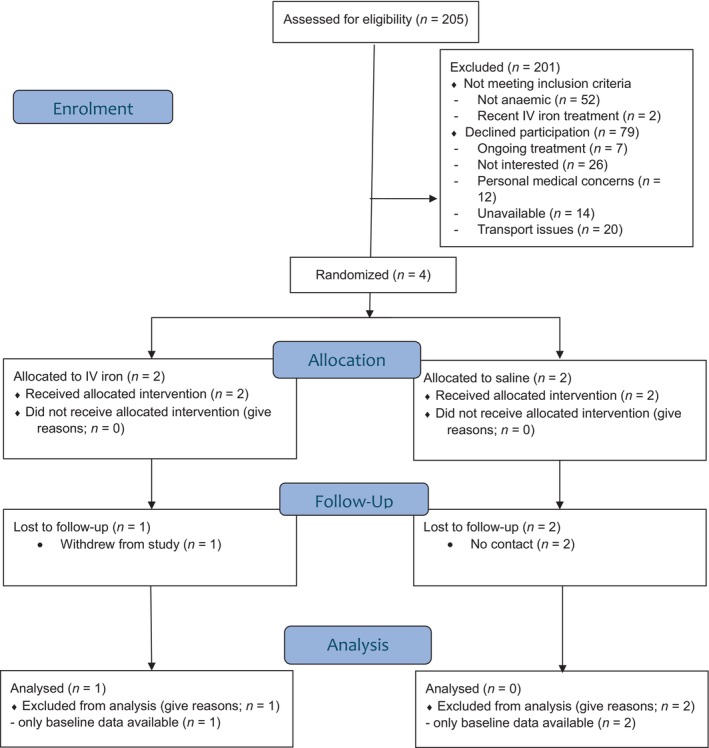
Consolidated Standards of Reporting Trials flowchart. Interventions include intravenous (IV) iron or saline (placebo).

Of 205 patients who had undergone eligible surgical procedures, screened between the 5 May 2023 and 31 August 2023, 137 patients were available for discussing recruitment to this study (Figure 1). Of the 137, 46 patients expressed interest in joining the study; however, ultimately, only four patients were consented and randomized.

Evaluation of recruitment hurdles for the 205‐patient cohort showed that 68 of these patients were not available for discussing recruitment, either due to being discharged, relocated or not in their room upon recruitment. Of the remaining 137 patients, 52 were not eligible to partake due to ineligible Hb concentration; 2 were not eligible to partake due to receiving intravenous iron; 7 declined participation due to ongoing treatment; 26 were not interested in the study; 12 declined due to personal medical concerns; 14 were not able to incorporate the study into their schedule and 20 were unable to attend the study due to issues with transport logistics (Table S1).

Of the four patients successfully recruited and randomized, one patient had undergone a hemicolectomy, one patient underwent laparotomy and repair of a paraduodenal hernia, one patient underwent laparotomy and repair of a ventral hernia and one underwent surgery for an abdominal aortic aneurysm. All four patients attended baseline assessments, with haematology and QoL captured for all patients, and three of the four patients were able to complete all baseline muscle function assessments. Upon randomization, two patients received intravenous iron and two patients received saline. Initial exercise classes were attended by two of the four patients, with one patient remaining in the trial by the second assessment at 8 weeks. As communication with drop‐out patients was lost, we have no insight into the factors that contributed to post‐randomization drop‐out. By the conclusion of the trial at 16 weeks, one patient successfully completed the trial; all remaining patients were lost to follow up. Given only one patient was successfully recruited and completed the trial in a 17‐week recruitment period, recruitment was deemed not feasible.

### Trial case study

As one patient successfully completed the study, the results are reported in the format of a case study. The recruited patient was a 78‐year‐old male who had undergone laparoscopic surgery to repair a ventral hernia. The participant reported no comorbidities, though he notably had undergone a recent hip replacement 4 weeks prior to the hernia operation, reducing the patient's mobility. The patient stayed in hospital for a total of 5 days with a Hb concentration of 116 g/L prior to discharge. Around 3 weeks after his operation, the patient underwent baseline assessments, with a Hb concentration of 126 g/L, ferritin of 114 μg/L and TSAT of 14% (Figure S1), suggesting the patient may have been experiencing iron deficiency in the setting of inflammation. The patient underwent randomization immediately after baseline assessments and consequently received 1000 mg of intravenous FCM.

QoL assessment (SF‐36v2) physical component score (PCS) was 27.4 and mental component score (MCS) was 27.6 at baseline, while EQ‐5D index was 0.848 and EuroQol visual analogue scale (EQ‐VAS) was 80 (Table S2). Maximum handgrip strength was 38 kg, which is above the standard threshold for identifying sarcopenic men (<30 kg) [10]. Upon assessment by the study exercise physiologist, the patient was not able to undergo the remaining functional assessments due to still being in recovery from recent hip replacement, but clearance for exercise was expected by Week 5 post‐operation.

Due to logistical difficulties, the participant rested until the Week 8 post‐discharge assessment. The participant exhibited an improvement in Hb concentration (134 g/L), ferritin (383 μg/L) and TSAT (29%) (Figure S1). Handgrip strength was 34.7 kg, the TUG test was completed in 13.6 s, sit‐to‐stand of 8 repetitions and six‐minute walk test (SMWT) of 310 m, achieving a gate speed of 0.86 m/s, above the sarcopenic threshold (<0.8 m/s) [10]. PCS increased to 33.4, and MCS increased to 32.9, while EQ‐VAS remained consistent at 80, and EQ‐5D index was 0.956 (Table S2).

Immediately after the assessment, the participant began twice‐per‐week exercise sessions. Exercises included a cycling warm‐up, weighted arm lifts (bicep curls, shoulder press, lateral and frontal raises), squats and weighted leg lifts (seated extension, hip flexion, leg curl, calf raise and hip abduction) with a focus on improving lower limb mobility. By the Week 12 post‐discharge assessment, Hb concentration was 138 g/L, ferritin was 360 μg/L and TSAT was 22% (Figure S1). Handgrip strength was 33.7 kg, TUG test was completed in 12.4 s, SMWT was 387 m and 9 repetitions were completed in the sit‐to‐stand test (Table S2). The patient reported a PCS of 38.1 and MCS of 40.1, displaying a positive improvement in the PCS score by 10.8 and similarly an improvement of 12.5 in the MCS component (Figure S2) EQ‐VAS remained consistent at 80, and similarly, EQ‐5D index was 0.848.

By the final assessment at 16 weeks post‐discharge, the participant had successfully attended all scheduled exercise sessions (16 sessions). Hb concentration was 139 g/L, ferritin was 331 μg/L and TSAT was 24% (Figure S1). Improvements were observed in muscle function tests: the TUG test was completed in 10.8 s, the SMWT was 410 m and 11 repetitions were completed in the sit‐to‐stand test, though handgrip strength remained consistent at 34.2 kg (Table S2). The PCS score remained consistent at 38.6, while the MCS declined to a score of 34.7 (Figure S2). The EQ‐VAS increased to 95, and similarly, the EQ‐5D index rose to 0.92.

## DISCUSSION

Despite the substantial prevalence of anaemia in the recruitment pool, recruitment to the trial was not feasible. Key limitations included a lack of appeal to patients, transport and schedule issues. Naturally, this trial demanded a substantial patient involvement, with two exercise sessions per week. This trial was also conducted onsite at a hospital where parking is limited and costly, which may have influenced trial uptake. The post‐operative recruitment timing may have negatively influenced trial uptake; however, unfortunately, pre‐operative recruitment was not logistically possible in this study. Though it was not feasible to recruit a substantial cohort in the current study, future work could explore the implementation of a remote (telehealth) exercise programme to reduce logistical hurdles to recruitment.

In the current case study, there was improvement in both mental and PCSs of the SF‐36v2 between baseline and Week 12 post‐discharge, while the patient's scores on the EQ‐5D‐5L suggested consistency in the index score and improvements in the visual analogue score by Week 16 post‐discharge. Notably the SF‐36v2 provides greater specificity and sensitivity to detect a change in these outcomes, due to the eight domains covered compared with the five domains covered through single questions in the EQ‐5D‐5L. Furthermore, the visual analogue score is purely determined by the patient's perception of their overall health, meaning improvements observed in the EQ‐VAS at the final study assessment (Week 16) may be more prone to the placebo effect.

From what was observed in the case study, there appeared to be improvements in muscle function by Week 16, with longer distances achieved in the SMWT, more sit‐to‐stand repetitions and quicker TUG tests. Notably, handgrip strength did not improve; however, the exercise programme was more optimized towards limb strength and endurance. Furthermore, the patient appeared to improve Hb concentration by 13 g/L from baseline to 16 weeks post‐discharge, though notably there was an improvement of 10 g/L observed between screening at discharge and the baseline assessment conducted 4 weeks later, prior to any intervention. Therefore, it is unclear whether the post‐treatment increase was influenced by iron therapy. Naturally, the results of the case study are limited due to the lack of comparison, and the influence of time spent recovering from surgery could account for the improvements observed.

To conclude, recruitment to a randomized controlled trial exploring the influence of iron therapy on muscle function after major abdominal surgery was not feasible.

## CONFLICT OF INTEREST STATEMENT

The authors declare no conflicts of interest.

## Supporting information


**Data S1.** Supporting information.

## Data Availability

The data that support the findings of this study are available on reasonable request to the corresponding author. The data are not publicly available due to privacy or ethical restrictions.
