# Supplementary material for: Feasibility of conducting a randomized controlled trial to explore the muscular influence of post‐operative intravenous iron treatment for anaemia after major abdominal surgery
Source: Vox Sang. 2025 Apr 22;120(7):678–82. doi: 10.1111/vox.70037 (PMC12286669; doi:10.1111/vox.70037)
Supplement: Supplementary file 1 — Data S1. Supporting information. [file VOX-120-678-s001.docx]

**Feasibility of conducting a randomised controlled trial to explore the muscular influence of post-operative intravenous iron treatment for anaemia after major abdominal surgery.**

Beth MacLean^1*^, Robert U. Newton^2,3^, Jayne Lim^1^ and Toby Richards^1,4^

1 University of Western Australia, Perth, Australia

2 Edith Cowan University, Perth, Australia

3 University of Queensland, Brisbane, Australia

4 University of East London, London, England

*Corresponding Author

[Beth.MacLean@research.uwa.edu.au](mailto:Beth.MacLean@research.uwa.edu.au)

The University of Western Australia,

35 Stirling Highway, Crawley

6009, Western Australia

**Supplementary Materials - Index**

| **Study Protocol** |  |  |
| --- | --- | --- |
| Detailed Protocol | *pag. 1 - 32* |  |
|  |  |  |
| **Supplementary Tables and Figures** |  |  |
| S.Table 1 | | *pag. 33* |
| S.Table 2 | | *pag. 34* |
| S.Figure 2 | *pag. 35* |  |
| S.Figure 3 | *pag. 36* |  |


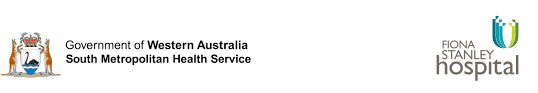

**Clinical Trial Protocol**

| **Short Title: Iron Deficiency Anaemia in Major Cardiac, Abdominal and VascuLar surgery patients and Effect on functional outcomes** |
| --- |

Full title: A two-arm, parallel-group double-blind randomised controlled trial of intravenous iron versus placebo in anaemic patients undergoing major cardiac, abdominal, or vascular surgery

Version 4.0
09^-^Feb-2023

Coordinating Principal Investigator: Professor Toby Richards

Sponsor: South Metropolitan Health Service

Trial registration: ClinicalTrials.gov, ANZCTR.org.au

# Protocol Signature Page

| **Short Title:** | Iron Deficiency Anaemia in Major Cardiac, Abdominal and VascuLar surgery patients and Effect on functional outcomes |
| --- | --- |
| **Protocol Version:** | V4.0 09-Feb-2023 |
| **Sponsor:** | South Metropolitan Health Service |

By signing this Protocol, the Investigator(s) acknowledges and agrees:

The Protocol contains all necessary details for conducting the study. The Investigator will

conduct this study as in accordance with this protocol and in compliance with Good Clinical Practice (GCP) and the applicable regulatory requirement(s).

| **Name:** | ________________________________________ |
| --- | --- |
| **Signature:** | ________________________________________ |
| **Date:** | ________________________________________ |

Table of Contents

[Protocol Signature Page 2](#_Toc184635279)

[List of abbreviations 5](#_Toc184635280)

[1. Abstract 6](#_Toc184635281)

[2. Administrative Information 8](#_Toc184635282)

[3. Introduction 9](#_Toc184635283)

[Background and Rationale [6a] 9](#_Toc184635284)

[4. Objectives [7] 11](#_Toc184635285)

[4.1 Primary Objectives 11](#_Toc184635286)

[4.2 Secondary Objectives 11](#_Toc184635287)

[5. Trial Design [8] 12](#_Toc184635288)

[6. Study Population [9] 12](#_Toc184635289)

[6.2 Eligibility Criteria [10] 13](#_Toc184635290)

[6.2.1 Inclusion criteria 13](#_Toc184635291)

[6.2.2 Exclusion criteria 13](#_Toc184635292)

[6.3 Recruitment [15] 13](#_Toc184635293)

[7. Study Procedures [13] 14](#_Toc184635294)

[7.2 Randomisation procedures 14](#_Toc184635295)

[7.2.1 Allocation [16a] [16b] [16c] 14](#_Toc184635296)

[7.2.2 Blinding [17a] 14](#_Toc184635297)

[7.2.3 Unblinding [17b] 15](#_Toc184635298)

[7.3 Perioperative group 16](#_Toc184635299)

[7.4 Recovery group 17](#_Toc184635300)

[7. Methods 18](#_Toc184635301)

[7.1 Routine clinical blood tests 18](#_Toc184635302)

[7.2 Core laboratory assessments 18](#_Toc184635303)

[7.2 Clinical assessments 19](#_Toc184635304)

[7.3 Quality of life assessments 19](#_Toc184635305)

[7.4 Functional assessments 19](#_Toc184635306)

[7.5 Criteria for discontinuing or modifying allocated interventions [11b] 21](#_Toc184635307)

[*7.6* Definition of end of trial 21](#_Toc184635308)

[7.7 Withdrawal of participants 21](#_Toc184635309)

[7.8 Relevant Concomitant Care Permitted or Prohibited During the Trial [11d] 22](#_Toc184635310)

[7.9 Post-trial care arrangements [30] 22](#_Toc184635311)

[8. Investigational Product [11a] 22](#_Toc184635312)

[8.1.1 Summary of known and potential risks and benefits 22](#_Toc184635313)

[8.2 Placebo 23](#_Toc184635314)

[8.3 Route of administration and dosage 23](#_Toc184635315)

[8.4 Preparation and labelling of Investigational Product 23](#_Toc184635316)

[8.5 Handling and Storage of Investigational Product 23](#_Toc184635317)

[9. Safety reporting [22] 23](#_Toc184635318)

[9.1 Adverse Event (AE) 23](#_Toc184635319)

[9.2 Serious Adverse Event (SAE) 24](#_Toc184635320)

[9.3 Suspected Unexpected Serious Adverse Reaction (SUSAR) 24](#_Toc184635321)

[9.4 Pregnancy 24](#_Toc184635322)

[10. Data Management [18a] 24](#_Toc184635323)

[10.1 Case Report Form 24](#_Toc184635324)

[10.2 Data management [19] 24](#_Toc184635325)

[10.3 Confidentiality [27] 25](#_Toc184635326)

[11. Statistical Considerations 25](#_Toc184635327)

[11.1 Outcomes [12] 25](#_Toc184635328)

[11.1.1 Primary Endpoint Change in Hb concentration from baseline to 90 days following randomisation 26](#_Toc184635329)

[11.1.2 Secondary Endpoints Laboratory: 26](#_Toc184635330)

[11.2 Sample size [14] 26](#_Toc184635331)

[11.3 Participant retention and complete follow-up [18b] 27](#_Toc184635332)

[11.4 Statistical Analysis [20a] 27](#_Toc184635333)

[11.5 Interim analyses [21b] 27](#_Toc184635334)

[12. Monitoring and Quality Assurance [23] 28](#_Toc184635335)

[13. Study Committees 28](#_Toc184635336)

[13.1 Project Management Group (PMG) [5d] 28](#_Toc184635337)

[13.2 Trial Steering Committee (TSC) [5d] 28](#_Toc184635338)

[13.3 Data Safety and Monitoring Committee (DSMC) [21a] 28](#_Toc184635339)

[14. Administrative Aspects 29](#_Toc184635340)

[14.1 Ethical Considerations 29](#_Toc184635341)

[14.2 Regulatory requirements 29](#_Toc184635342)

[14.3 Protocol amendments [25] 29](#_Toc184635343)

[14.4 Publication policy [31a] 29](#_Toc184635344)

[14.5 Archiving 29](#_Toc184635345)

[References 30](#_Toc184635346)

# List of abbreviations

| AE | Adverse Events |
| --- | --- |
| CFS | Clinical Frailty Scale |
| CRF | Case Report Form |
| CRP | C-Reactive Protein |
| DAH | Days alive and out of hospital |
| DSMC | Data Safety and Monitoring Committee |
| EPO | Erythropoietin |
| ESR | Erythrocyte Sedimentation Rate |
| EQ-5D-5L | European quality of Life – 5 dimensions – 5 levels |
| GCP | Good Clinical Practice |
| GMP | Good Manufacturing Practice |
| Hb | Haemoglobin |
| HIF | Hypoxia-inducible Factor |
| HREC | Human Research Ethics Committee |
| IP | Investigational Product |
| ISF | Investigator Site File |
| I.V. | Intravenous |
| MCV | Mean Corpuscular Volume |
| PI | Principal Investigator |
| PH | Prolyl Hydroxylase |
| PICF | Participant Information Sheet and Consent Form |
| PMG | Project Management Group |
| QoL | Quality of Life |
| RBC | Red Blood Cell |
| RDW | Red cell Distribution Width |
| SAE | Serious Adverse Event |
| SOP | Standard Operating Procedure |
| SST | Serum separator tube |
| STFR | Soluble transferrin receptor |
| SUSAR | Suspected Unexpected Serious Adverse Reaction |
| TGA | Therapeutic Goods Administration |
| TSat | Transferrin Saturation |
| TSC | Trial Steering Committee |

# Abstract

**Background**

Iron deficiency is the commonest nutritional deficiency globally, affecting 2 billion people and is the leading cause of anaemia. Iron deficiency anaemia makes people tired and unwell as well as impacting physical function, it is a WHO top 10 leading cause for disability. (1, 2) Anaemia is common in patients undergoing major surgery and associated with worse patient outcomes (3-5) and increased post operative mortality. (3, 5-7)

The aetiology of anaemia in the surgical patient is multifactorial; it can be related to co-morbidities and chronic disease, the illness or problem for which the patient is undergoing surgery, blood loss at operation or secondary to surgical inflammation. However, the mechanism of anaemia in surgical patients is not clear. Hepcidin, the master regulator of iron homeostasis, impacts iron absorption and cellular transport by regulation of the main iron transport protein (Ferroportin). Upregulation of Hepcidin by Cytokines (particularly IL-6) leads to a functional iron deficiency as iron transport in the reticuloendothelial system is impaired due to sequestration of iron into macrophages. If prolonged this can lead to anaemia of chronic disease. Conversely, blood loss inhibits hepcidin to promote iron absorption and transport to the bone marrow, but post operatively it is unclear how recovery of anaemia and hypoxic drive for erythrogenesis is impacted by this inflammatory modulation.

The aim of this trial is to explore the mechanisms of iron deficiency over the perioperative time period in patients undergoing cardiac, abdominal and vascular surgery and the response to intravenous iron therapy that bypasses the normal Hepcidin mediated iron pathways. Secondary aims include to assess the efficacy of intravenous iron and feasibility of measuring potential effects on patients’ physical recovery.

**Methods**

AMBLE will be a prospective, parallel-group, double blinded, 2 by 2 factorial, randomised, multi-centre trial designed to investigate iron deficiency in cardiac, abdominal, and vascular surgery patients. Potential participants will be identified through normal clinical pathways for surgery and anaesthesia such as surgical outpatient and preoperative clinics, surgical admissions, or inpatient wards. The Perioperative group will assess the mechanisms of iron deficiency and response to intravenous iron therapy in the perioperative period. The Recovery group will assess feasibility to test patients physical and quality of life recovery in the postoperative setting and assess if this differs between iron deficient and iron replete patients.

Two separate groups of patients will be assessed:

- The Perioperative group of patients included if about to undergo major cardiac, abdominal and vascular surgery to assess the mechanisms of iron deficiency in the perioperative period.
- The Recovery group of patients included 4 weeks after hospital discharge following major cardiac, abdominal and vascular surgery to assess recovery of physical function and anaemia.

Following screening, participants who provide informed consent will undergo baseline blood tests, quality of life (QoL) questionnaires and functional assessments. Following this, participants will be randomised to either an intravenous iron administered either directly post-operatively in the Perioperative group or 4 weeks post operatively in the Recovery group, or placebo.

Follow up assessments will occur:

- The Perioperative group at Days 1, 2, 4, 8 and 2 weeks then weeks 4, 8, 12 and 16.
- The Recovery group weeks 4, 8, 12 and 16.

**Discussion**

The outcomes of this research will generate a better understanding of the mechanism of iron deficiency anaemia in surgical patients as well as how this affects post operative recovery. By better understanding the aetiology of iron metabolism in surgery we can determine what intervention (iron +/- EPO) may improve patient outcomes and the administration timing thereof. As a result, more informed decisions will be made regarding iron supplementation and therapy in these patients.

**Keywords** Vascular Surgery, Abdominal surgery, Anaemia, functional outcome, Critical Limb Ischemia, Major Limb Amputation, Major Adverse Cardiac Events

# Administrative Information

This protocol has been developed in accordance with Standard Protocol Items Recommendations for Intervention Trials (SPIRIT) 2013 guidelines and reported using CONSORT guidelines. Numbers in square brackets refer to SPIRIT checklist item numbers. The order of items was modified to group similar items.

| Full Title [1] | A two-arm, parallel-group double-blind randomised controlled trial of intravenous iron versus placebo in anaemic patients undergoing major cardiac, abdominal, or vascular surgery | |
| --- | --- | --- |
| Short Title | AMBLE: Iron Deficiency **A**naemia in **M**ajor Cardiac, A**b**dominal and Vascu**L**ar surgery patients and **E**ffect on functional outcomes |  |
| Protocol version [3] | Version 4.0 09-Feb-2023 | |
| Funding [4] | This trial is supported by the Michael Lawrence Brown fund. | |
| Author details [5a] | Professor Toby Richards  Dr. Elizabeth Magdelin Hines Dr. Ines Clement  Beth McClean | |
| Sponsor [5b] | South Metropolitan Health Service  14 Barry Marshall Parade  Murdoch WA 6150 | |
| Trial registration [2a & b] | ClinicalTrials.gov and ANZCTR.org.au prior to recruitment | |

# Introduction

## Background and Rationale [6a]

**Anaemia worsens outcomes for surgical patients:**

Anaemia affects nearly a quarter of the world (6) and is common in surgical patients with a third of patients presenting with preoperative anaemia and three quarters of patients discharged from hospital with anaemia (7). The World Health Organisation defines anaemia as an insufficient circulating red cell mass, with a haemoglobin (Hb) concentration of < 130 g.l−1 for men and < 120 g.l−1 for women (6).

Perioperative anaemia is associated with increased postoperative complications and delayed patient recovery leading to increased post-operative morbidity and mortality (6-12) Anaemia also leads to an increased use of allogeneic blood transfusions (12-15), which is an independent risk for poorer patient outcomes (15-18).

The most common cause of preoperative anaemia is iron deficiency, which can be caused by reduced or impaired dietary iron absorption, chronic blood loss, or disruption of normal iron metabolism due to comorbidities or inflammation, aetiologies commonly seen in patients undergoing major abdominal surgery, which causes an increase in hepcidin production resulting in functional iron deficiency (18-20) and reduced red cell production.

**Clinical trials on intravenous iron:**

The use of intravenous iron has become a normal treatment for anaemia in Australia and supported by international guidelines. (21, 22) We investigated the efficacy of intravenous iron to treat anaemia (23) suggesting efficacy to increase haemoglobin. This was confirmed in our multicentre cohort study of patients before Cardiac and Vascular Surgery which showed efficacy to increase haemoglobin levels but with no patient effect (24, 25). In large multicentre PREVENTT RCT we randomised 487 patients at 46 centres to intravenous iron or placebo 10-42 days before major abdominal surgery. (26) Unexpectedly there was no impact on blood transfusion (29% vs. 28%; p = 0.92) or postoperative complications (24 [11%] vs. 22 [9%]; RR 0.89, 95% CI 0.52 to 1.55; p = 0.69), length of hospital stay (median [IQR]: 9 [7–14] days vs. 9 [5–14] days; p = 0.14), or mortality rate (1% vs. 1%; p = 1.0) These findings were unaffected by subgroup analysis (age, gender, [Hb]) or, on reanalysis, by markers for iron deficiency (ferritin, Tsats).(27)

The NOVEL FINDING in PREVENTT was that the greatest impact from intravenous iron was seen after surgery with a significant rise in [Hb] at 8 weeks (MD: 10·7 g/L; 95% CI, 7·8- 13·7). This efficacy of intravenous iron was associated with a reduced readmission rate to hospital for post operative complications (21.8% to 13.8%; relative risk reduction 29.5-39.4%) and reduced additional bed stay (877 to 299 days).

We confirmed this finding in a large multicentre prospective RACS CTANZ audit of 2730 patients at 56 hospitals in Australia and New Zealand. Preoperative anaemia was seen in 28% of patients undergoing major surgery and associated with an increased risk of transfusion, postoperative complications, reoperation, MET calls, and a longer length of hospital stay (all p<0.001). Importantly, Postoperative anaemia was common (59.2%, 1186/2003), and associated with a higher rate of readmission (13.5% vs. 7.6%, p<0.001). (MJA 2022 in press). (27)

**Understanding the mechanism of iron deficiency in surgical patients:**

Surgical anaemia can be due to chronic disease, blood loss at operation or secondary to surgical inflammation. Cytokines (particularly IL-6) upregulate hepcidin, the master regulator of iron homeostasis, which prevents iron transport leading to failure of dietary iron absorption, and sequestration of iron within macrophages. (28, 29) This leads to functional iron deficiency and subsequently anaemia of chronic disease.

The efficacy of intravenous iron therapy may be impacted by the balance between the inflammatory modulation of iron sequestration and bone marrow suppression with the hypoxic drive for erythrogenesis. Although efficacy of intravenous iron was shown in the IRONMAN trial, we performed in patients following ICU and the PREVENTT trial in preoperative patients, the one size all approach may not be precise. (30, 31) Consequently, in the surgical pathway the optimal timing and modality (intravenous iron +/- EPO) is unknown. Further, with development of disease modifying agents (anti-IL6 therapy or Hepcidin antagonists) and small molecule anaemia therapies with Hypoxia-inducible factor (HIF) prolyl hydroxylase (PH) enzyme inhibitors there is a need to understand the fundamental basis and aetiology of iron deficiency anaemia in the surgical patient.

**Hypothesis:**

Intravenous iron can support erythrogenesis despite post-operative inflammation and improve patient muscle function quality of life and post operative recovery.

# Objectives [7]

## 4.1 Primary Objectives

1. To investigate the pattern and causality of iron deficiency in patients before, during and recovering from major surgery.
2. To investigate the effects of iron deficiency and treatment with intravenous iron and on the functional and mental wellbeing patients during and recovering from major surgery.

## 4.2 Secondary Objectives

Investigate the effect of iron deficiency and anaemia in relation to:

1. Laboratory Outcomes:
   1. Routine Haematology markers of: Hb, MCV, RDW
   2. Routine Iron markers of: Ferritin & transferrin saturations
   3. Experimental Iron markers including: STFR, Hepcidin, Erythroferrone and EPO
   4. Experimental Inflammatory markers including: CrP, IL6 and other cytokines
   5. Proteomic and metabolomic profiles.
2. Mental and physical recovery after surgery
   1. Complications as measured by the Clavien Dindo Scale
   2. Length of hospital stay
   3. Re-operations
   4. Unplanned re-admission to hospital for complications
   5. Days Alive and at Home (DAH) 30 & 90
   6. Clinical frailty score and activities of daily living (Nottingham extended ADL scale)
   7. Sarcopenia as assessed by quantitative skeletal muscle analysis and quantative skeletal muscle changes post intervention (where imaging available)
   8. Functional assessments as assessed by Short Physical Performance Battery; 4 metre gait speed, five time sit-stand test, balance test. Maximal handgrip strength, time to get up and go, or 6-minute walk test
   9. Quality of life measured by SF-36v2 health survey, EQ-5D-5L questionnaire and the POMS questionnaire
3. Adverse events / serious adverse events
   1. Assessment of major adverse cardiac events
   2. Assessment of major adverse limb events
   3. Discharge destination

# 5. Trial Design [8]

Multicentre, prospective, parallel-group, double blinded, 2 by 2 factorial, randomised controlled trial comparing intravenous iron or placebo administration in anaemic patients undergoing major cardiac, abdominal, or vascular surgery.

Perioperative group: Intravenous iron or placebo will be administered immediately post-operatively.
Recovery group: Intravenous iron or placebo will be administered 4 weeks post-operatively.

All patients will be followed for 16 weeks from date of randomisation.

## **6. Study Population [9]**

This study will include patients undergoing cardiac surgery (coronary bypass or cardiac valve surgery), vascular surgery (patients with critical limb threatening ischaemia undergoing open and endovascular revascularisation procedures), and abdominal surgery (patients undergoing major open or laparoscopic procedures). All groups where iron deficiency and anaemia have been identified to be high risk and impact patient outcomes. The comparators will be the interventions before and after randomisation of treatment.

- The Perioperative group of patients included if about to undergo major cardiac, abdominal and vascular surgery will be consented prior to surgery (See Section 7.2)
- The Recovery group of patients included 4 weeks after hospital discharge following major cardiac, abdominal and vascular surgery will be consented post-operatively (See Section 7.2)

The Perioperative group will assess the mechanisms of iron deficiency and response to intravenous iron therapy in the perioperative period. The Recovery group will assess the feasibility to test patients physical and quality of life recovery in the post-operative setting and assess if this may differ between iron deficient and iron replete patients.

## 6.2 Eligibility Criteria [10]

### 6.2.1 Inclusion criteria

Patients who meet the following criteria at the start of treatment are eligible for the study:

1. Adults (≥18 years)
2. Undergoing planned or unplanned (elective/expedited or emergent) cardiac surgery (bypass or valve), abdominal surgery (open or laparoscopic) or vascular surgery (open or hybrid) of the lower limb or major amputation (above and below knee)
3. Anaemia (Hb <130g/L in males and <120g/L) in females
4. Willing and able to undergo follow-up visits

6.2.2 Exclusion criteria
Patients who, at the start of treatment, meet any of the following criteria are not eligible for the study:

1. Blood transfusion at operation or blood transfusion in previous 3 months
2. Erythropoietin or intravenous iron in the previous 4 weeks
3. Known hypersensitivity to (ferric carboxymaltose or equivalent) or its excipients
4. Active infection on therapeutic antibiotics
5. Known chronic liver disease
6. Known other cause for anaemia (eg. untreated B12 or folate deficiency or myelodysplasia)
7. Known family history of haemochromatosis or TSATS >50%
8. Pregnancy or lactation
9. Unable to provide written informed consent

All patients will follow the standard care pathways and thresholds for anaemia management, typically red blood cell (RBC) transfusion for Hb <70g/L.

## 6.3 Recruitment [15]

This is a hospital-based study at sites in Australia and the United Kingdom. Participating hospital sites may enrol patients into one or more of the clinical cohorts. Further locations may be added. Patients will be identified through the normal clinical patient pathways for surgery and anaesthesia such as surgical outpatient and preoperative clinics, surgical admissions, or inpatient wards. **As part of normal clinical assessment for major surgery, all patients will be screened for anaemia. Following** **review and discussion with the treating** clinical **team, patients** will be invited to **participat**e **in the trial.**

# 7. Study Procedures [13]

7.1 Informed consent [26a][26b]

Written informed consent will be obtained prior to performing any trial-specific procedure by the investigator or delegate. Participants must be given written and verbal information, in lay terms, of the aims, methods, anticipated benefits and potential hazards, and given sufficient time to consider whether they wish to participate. The participants must be notified that participation is voluntary, and that they are free to discontinue treatment or withdraw consent from the study at any time without any disadvantages for their future care and treatment. If the participant agrees to participate in the trial, they will be asked to initial, sign and date the latest version of the Participant Information Consent Form (PICF). The Investigator or delegate will then sign and date the form. A copy of the PICF will be given to the participant, a copy will be filed in the medical notes.

Patients will be invited at the time of study enrolment to provide optional written informed consent for donation of blood sample for future biological or translational sub-studies
for future research use, pending relevant ethics and governance approval.

## 7.2 Randomisation procedures

### 7.2.1 Allocation [16a] [16b] [16c]

Random allocation will be performed using a computer-generated code, access via a web-based service. Randomisation will be performed by the unblinded study personnel delegated this responsibility by the principal investigator as evidenced by documentation in the delegation log. The blinded staff will not have access to the randomisation system and will therefore remain blinded to the treatment allocated. Each unblinded study personnel will be trained in the use of the web-based service before they are provided with their own individual password and pin code to access the service.

### 7.2.2 Blinding [17a]

As active and placebo fluids cannot be matched in appearance, unblinded study personnel (unblinded nurse or hospital pharmacist) will be responsible for the preparation of the study drug. The study drug will be shielded from vision (light protection bags) and administered through black tubing. The unblinded study personnel will also dispose of the administration kit in a concealed way.

### 7.2.3 Unblinding [17b]

Treatment unblinding will only occur when there are legitimate clinical concerns where the appropriate management of the participant necessitates knowledge of the treatment allocation. All cases of emergency unblinding will be thoroughly documented and reported to the data safety monitoring board (DSMB). After all participants have completed the study, participants will be notified which arm of the study they took part in.

## 7.3 Perioperative group

| **Perioperative group** | | | | | | | | | |
| --- | --- | --- | --- | --- | --- | --- | --- | --- | --- |
| **Perioperative group (Amputation)** | | | | |  | | | | |
| **Procedures** | **Pre Op (- 4 weeks to 0 day)** | **Baseline Day of surgery** | **Post Op Days 1,2,4** | **Discharge or day 8** | **2 weeks (± 3 days)** | **4 weeks (± 1 wks)** | **8 weeks (± 2 wks)** | **12 weeks (± 2 wks)** | **16 weeks (± 2 wks)** |
| **Informed consent** | X |  |  |  |  |  |  |  |  |
| **Inclusion/ exclusion criteria** | X |  |  |  |  |  |  |  |  |
| **Medical History** | x |  |  |  |  |  |  |  |  |
| **Concomitant medications** | x |  |  | x | x | x | x | x | x |
| **Clinical frailty scale** | x |  |  | x | x | x | x | x | x |
| **Routine clinical**  **blood tests** | x |  | x | x | x | x | x | x | x |
| **Core Laboratory assessments** |  | x | x | x | x | x | x | x | x |
| **Randomisation** |  | x |  |  |  |  |  |  |  |
| **Study medication administration** |  | x |  |  |  |  |  |  |  |
| **Adverse events** |  | x | x | x | x | x | x | x | x |
| **Quality of Life assessments** |  |  |  | X | x | x | x | x | x |
| **Functional assessments** |  |  |  | x | x | x | x | x | x |

**7.3.1 Schedule of assessments**

**7.3.2 Study Visits**

Patient assessments will start on admission to hospital on the day of surgery (Day 0). Patients will be randomly assigned to receive either intravenous iron or placebo at skin closure in operating theatres administered by the anaesthetics team, if this is not possible for logistical reasons, the patient will receive the one-off infusion in recovery as per local policy.

During inpatient admission, results from routine blood tests measured at day 1, 2, 4 and 8 (if still an inpatient at this time) will be recorded on the CRF. These will be performed as part of the routine clinical post-operative care. An additional SST blood sample (6-8mL or 1.5 teaspoons) will be collected at the time of the routine blood draws for core laboratory analysis.

Subsequent follow up in outpatient at weeks 2, 4 (+/- 1), 8 (+/- 2), 12 (+/- 2), 16 (+/- 2) from the operation date. Functional and quality of life assessments will be collected at those time points.

## 7.4 Recovery group

**7.4.1 Schedule of assessments**

| **Procedures** | **Baseline**  **(4 weeks post discharge)** | **4 weeks (± 1 wks)** | **8 weeks (± 2 wks)** | **12 weeks (± 2 wks)** | **16 weeks (± 2 wks)** |
| --- | --- | --- | --- | --- | --- |
| **Informed consent** | x |  |  |  |  |
| **Inclusion/ exclusion criteria** | x |  |  |  |  |
| **Medical History** | x |  |  |  |  |
| **Concomitant medications** | x |  |  |  |  |
| **Clinical frailty scale** | x | x | x | x | x |
| **Routine clinical**  **blood tests** | x | x | x | x | x |
| **Core Laboratory assessments** |  | x | x | x | x |
| **Randomisation** |  | x |  |  |  |
| **Study medication administration** |  | x |  |  |  |
| **Adverse events** |  | x | x | x | x |
| **Quality of Life assessments** | x | x | x | x | x |
| **Functional assessments** | x | x | x | x | x |

**7.4.2 Study Visits**

Patient assessment will start four weeks post discharge from hospital. Following baseline assessment, patients will be randomly assigned to receive either intravenous iron or placebo in the outpatient setting.

Participants will be required to attend up to 4 occasions for functional, quality of life assessment, and blood sampling. Where possible these visits will coincide with normal clinical follow up after discharge from hospital and it is envisaged only two additional visits will fall outside routine clinical care. The blood tests, QoL questionnaires and functional assessments will be undertaken at the same clinic visit.

# 7. Methods

## 7.1 Routine clinical blood tests

These, taken as part of normal clinical care, will be processed at the Local Laboratories and include (but not exclusively): FBC, UE, Iron Studies, B12, Folate and CRP.

## 7.2 Core laboratory assessments

One single tube (6-8mL or 1.5 teaspoons) will be collected and processed into four to six aliquots at each site (further details can be found in the Manual of Procedures).These will be centralised on block to the research team (Harry Perkins Institute of Medical Research South). Research samples will be stored in a de-identified manner at the study site in a -80 freezer at local sites. They will be shipping in bulk to the central store (Laboratory freezer owned by the Toby Richards research team located at Harry Perkins Institute of Medical Research - South).

Laboratory analyses will include but not exclusively markers of erythrogenesis (EPO, Erythroferrone), markers of iron metabolism (Ferritin, Tsats, sTFR), markers of inflammation (CrP, IL6, IL22), markers of phosphate metabolism (Vit D, FGF23, Phosphate) and phenomic and metabolomic analyses.

For the purpose of this current trial, one de-identified aliquot will be shipped to The Lakhal-Littleton Group, Department of Physiology, Anatomy & Genetics, Oxford University, UK for iron metabolism analyses (hepcidin and ferroportin).

Remaining serum and aliquots will be held in the research team -80 freezer for future use. Access to samples for additional analyses will be governed by a trial steering committee comprising the clinical lead investigators and scientific investigators for this study. Where possible and within the constraints of international law and specific requirements of local ethical and institutional management approvals.

## 7.2 Clinical assessments

The time schedule for the assessments and the time windows allowed are provided in the flow chart in Section 6.4 and 6.5. All patients must be followed according to protocol, clinical status permitting, whether they receive their allocated trial treatment or not (unless the patient withdraws their consent for follow-up).

**Clinical frailty score**

Clinical Frailty Scale (CFS) will be used to assess frailty of patients enrolled in the trial. CFS is used to predict health outcomes that are significantly associated with mortality, morbidity, functional decline, mobility, and cognitive decline. It uses a 9-point scale (very fit -severely terminally ill).

## 7.3 Quality of life assessments

**SF-36v2**
SF-36v2 is a health survey that measures the functional health and well-being from the point of view of the patient. It can be used across age, disease, and treatment groups, in contrast to a disease-specific health survey which focuses on a particular disease or condition. Version 2 will be used and is modified for use in Australia.

**European Quality of Life-5 Dimensions-5 Levels (EQ-5D-5L)**The Eq-5D-5L is a brief, utility- based HRQoL instrument. It consists of a health descriptive system and a visual analogue scale (EQ-VAS) for respondents to self-classify and rate their health on the day of administrating of the instrument.

## 7.4 Functional assessments

It is not envisaged that all patients will undergo all these tests at all participating hospital sites. Some sites may be able to undertake a 6MWT and others not. Similarly, this may not be feasible in some patients (vascular). The feasibility to undertake one or all these tests is part of the outcome evaluation of the trial. i.e. what functional tests can routinely and accurately be performed in the post operative and recovery period after surgery?

**Grip Strength**Participants will be assessed using a calibrated JAMAR dynamometer. Each hand will be assessed twice and the greater of the two forces will be recorded and correlated to a rating system (excellent, very good, etc.).

**Six-minute walk test**

Patient will be assessed on a straight 30 metre track that they will lap for 6 minutes to objectively measure their functional ambulatory capacity throughout study. A resting electrocardiogram done during the previous 6 months will be reviewed prior to testing.

**Absolute contraindications:**

1. Unstable angina during the previous month
2. Myocardial infarction during the previous month

**Relative contraindications:**

1. Resting heart rate > 120
2. Systolic blood pressure > 180 mm Hg
3. Diastolic blood pressure > 100 mm Hg

**Sit-to-stand (STS) test**

The sit-to-stand test measures a patient’s leg strength and endurance by having them stand up from a sitting position repeatedly over the course of 30 seconds.

**The Timed Up and Go test (TUG)**

The TUG test measures a patient’s mobility, balance, walking ability and the fall risk. It uses the times a patient standing from a chair with no arms, walking 5 metres and returning to sit down.

##

## 7.5 Criteria for discontinuing or modifying allocated interventions [11b]

Adverse events occurring in connection with the administration of study medication will be recorded. In the event of a participant having an allergic reaction or signs of intolerance during study drug administration, the investigator must immediately stop the study treatment and notify the principal investigator. If the treatment is stopped for other reasons and the patient is willing the treatment can be restarted where possible (as per local practice).

## *7.6* Definition of end of trial

The end of the study is defined as the date that the last patient has completed his/her last study visit, or one year following randomisation of this last patient.

***Premature termination of the entire study***

The sponsor has the right to terminate the study at any time. Reasons which may require termination include the following:

- Recommendation from the DSMC.
- New toxicological or pharmacological findings or serious adverse events invalidate the earlier positive risk-benefit-assessment.
- The incidence and/or severity of adverse events in the study indicate a potential health hazard caused by treatment with the study medication.

## 7.7 Withdrawal of participants

A patient may decide to withdraw from the study at any time without prejudice to their future care. A patient may withdraw from the follow-up visits or they may withdraw their consent for any data collected to be used. Patients will be encouraged to allow data and samples that have been collected before withdrawal to be used in the analyses. However, if consent to use data/samples is also withdrawn, then these will be discarded. Patients withdrawing from the study will continue to be followed-up by their local team.

Patients who have consented and been randomised but do not receive the investigational product will stay on the study and will be followed up as in line with this protocol unless patient consent is withdrawn.

If a patient's treatment was stopped before being fully given for any reason the patient will be followed up as in line with this protocol unless consent is withdrawn.

Patients who withdraw from the trial will not be replaced, but their data will be included based on the intention-to treat principle (unless they withdraw consent for their data to be used).

## 7.8 Relevant Concomitant Care Permitted or Prohibited During the Trial [11d]

During the trial, the study team will ask participants to continue their normal activities, in particular:

1. Avoid major changes to diet and or diet manipulation strategies
2. Avoid iron supplementation

All medications being continued by a patient on enrolment and all medications given in addition to the study medication during the study are regarded as concomitant medications and will be documented by the patients in the patient diaries (these details will be transcribed into the eCRF), and in the patient’s medical records. All changes to concomitant medication taken from enrolment until completion of the study will also be recorded (including changes in dose, change in formulation, starting or stopping medication).

## 7.9 Post-trial care arrangements [30]

There will be no post-trial treatment arrangements as the treatment is a single dose. At completion of the study, participants will continue clinical follow up as necessary or be discharged from clinical follow up as per standard of care, this decision will be made by their usual clinician.

# 8. Investigational Product [11a]

8.1 Description of Investigational Product
The investigational product is intravenous iron preparation. Ferric carboxymaltose (Ferrinject) or iron isomaltoside (Monofer®) will be used as per local practice. For further information please refer to the summary of product characteristics.

### 8.1.1 Summary of known and potential risks and benefits

The side effects of the intravenous iron are minimal: the common side effects (>=1/100, <1/10) are headache, dizziness, hypertension, nausea, alanine aminotransferase increased, hypophosphataemia and injection site reactions, as detailed in the summary of product characteristics.

## 8.2 Placebo

Medication name: NaCl (normal saline)

Trade name: NaCl

Active ingredient: NaCl (normal saline)

Dosage form: 0.9% w/v NaCl as sterile solution in water for injection

Excipients: Water

Strength/Packaging: 100 mL container with 100 mL normal saline

Manufacturer: As per local hospital supplier

## 8.3 Route of administration and dosage

IV administration of trial drug will be given by slow IV push (Ferrinject®) or infusion over 15 - 30 minutes (Monofer®). Administration will be given in accordance with local practice for the use of intravenous iron.

Participants should be closely monitored as per local hospital standard care protocol throughout administration of the treatment.

## 8.4 Preparation and labelling of Investigational Product

Packages of investigational product will be labelled with storage conditions (store below 30°C), batch number and expiry date, as per the manufacturer. Labelling of the investigational product will comply with Therapeutic Goods Administration (TGA) code of Good Manufacturing Practice (GMP) for investigational products, Annex 13, section 17 in Australia, or applicable national and/or local requirements for international sites.

## 8.5 Handling and Storage of Investigational Product

Investigational product will be stored below 30°C until use in accordance with manufacturer’s instructions. The site investigator or delegate must maintain an adequate record regarding the administration of all investigational product within the trial. Accountability documentation will also reside with the site investigator and be retained in the Trial Master File at the coordinating centre.

# 9. Safety reporting [22]

9.1 Adverse Event (AE)

An AE is any untoward medical occurrence in a patient temporarily associated with the use of a medicinal product, whether or not considered related to the medicinal product. An AE can therefore be any unfavourable and unintended sign (including an abnormal laboratory finding), symptom, or disease (new or exacerbated) temporally associated with the use of a medicinal product, whether or not considered related to this medicinal product.

All adverse events will be captured and reported as per the CONSORT guidelines. Detailed description can be found in the procedures manual.

9.2 Serious Adverse Event (SAE) 
A SAE is any untoward medical occurrence that at any dose:

- Results in death,
- Is life-threatening,
- Requires hospitalisation or prolongation of existing hospitalisation,
- Results in disability/ incapacity,
- Congenital anomaly/birth defect,
- Other important medical events which, in the opinion of the investigator, are likely to become serious if untreated, or as defined in the protocol

## 9.3 Suspected Unexpected Serious Adverse Reaction (SUSAR)

A SUSAR is any SAE that is both suspected to be related to the study treatment and is unexpected (i.e., not consistent with applicable product information).

## 9.4 Pregnancy

Should a participant become pregnant whilst undertaking the trial, the pregnancy and resulting child will be followed up for a period of no less than 12 months.

# 10. Data Management [18a]

## 10.1 Case Report Form

A Case Report Form (CRF) will be completed for each participant. Participants will be referred to in the CRF by their initials, study number and the date of the visit for identification. All participating hospital sites must store any paper-based forms in accordance with GCP guidelines.

## 10.2 Data management [19]

Data will be recorded in an electronic CRF. The AMBLE Research Electronic Data Capture (REDCap) database is hosted on by Fiona Stanley Hospital, South Metropolitan Health Services behind a firewall with two factor authentication for administrators using a modern web browser. Access to the database at each participating hospital site will be secure and password protected and managed by the Data Manager. Access to the REDCap database at each participating hospital site will be granted after documented training. All information collected on the eCRF will have identifying information removed.

All original CRF will be securely archived at the participating hospital site for a minimum of 15 years and in accordance with applicable legislation.

Data will be extracted from the system by exporting the database table as CSV text files or another suitable format. Analyses will be conducted by the trial statistician in a statistical package (such as STATA) after importing the database tables. For unblinded analyses these files may be combined with the CSV file exported form the unblinded randomisation system.

## 10.3 Confidentiality [27]

A deidentifying study number will be assigned to each participant. This code will be maintained in a secure REDCap database accessible only to the Investigators. Others working with the data will only have access to the study number.

The study will be conducted in accordance with applicable Privacy Acts and Regulations. All information will be stored securely at the participating hospital sites and will only be accessible to people directly involved with the study. Personal data identifying tpatients will be held securely at the participating hospital sites in a locked filing cabinet requiring authorised key pass access for research team.

# 11. Statistical Considerations

## 11.1 Outcomes [12]

*Primary Outcome*

Results from the Full Blood Count for Haemoglobin levels will be collected from local laboratories

*Secondary Outcomes*

In hospital, post-operative complications measured by Clavien-Dindo scale will be collected prospectively by the research team during planned visits for venesection. Length of hospital stay and 30-days outcomes of; reoperation, re-admission to hospital or mortality will be documented locally and confirmed at the 2 and 4 week follow up visits. Any AE / SAE will be similarly recorded. Follow up visits will document Quality of life and functional assessments by the research staff.

### 11.1.1 Primary Endpoint Change in Hb concentration from baseline to 90 days following randomisation

### 11.1.2 Secondary Endpoints Laboratory:

1. Change in Routine Iron markers
2. Changes in Experimental Iron markers
3. Changes in proteomic and metabolomic profiles.
4. Mental and physical recovery after surgery
5. Complication rate at 30 days post operation
6. Length of hospital stay
7. Re-operations
8. Unplanned re-admission to hospital for complications
9. Days Alive and at Home (DAH) 30 & 90
10. Change in clinical frailty score and activities of daily living (Nottingham extended ADL scale)
11. Changes in Sarcopenia
12. Changes in functional assessments
13. Changes in Quality of life

## 11.2 Sample size [14]

There is no formal sample size calculation and a total trial population of 240 patients is proposed. These numbers may reduce following review of interim analyses and feasibility assessment planned at 6 monthly intervals.

The Perioperative group is mechanistic, and it is envisaged to include 40 patients in each of the three clinical cohorts with 20 patients randomised to intravenous iron and 20 patients randomised to placebo (Cardiac, Vascular and Abdominal Surgery) totalling 120 patients.

The Recovery group is exploratory, and it is envisaged to include 40 patients in each of the three clinical cohorts with 20 patients randomised to intravenous iron and 20 patients randomised to placebo (Cardiac, Vascular and Abdominal Surgery) totalling 120 patients.

## 11.3 Participant retention and complete follow-up [18b]

Participants will be engaging in an outpatient clinic care. However, in our pilot audit, we had significant difficulty completing follow up with patients who lived in rural Western Australia due to the travel distance in order to attend more frequent follow up appointments. Participants will be given the opportunity to complete trial follow up on their scheduled medical or surgical outpatient clinic appointment days where possible.

## 11.4 Statistical Analysis [20a]

Analyses will be based on all randomised participants. Reporting of research findings will be in accordance with CONSORT guidelines. Baseline demographic data and other participant characteristics will be expressed as means and standard deviations for continuous variables and counts and percentages for categorical variables, with 95% confidence intervals provided for estimates of interest where appropriate. The differences in baseline characteristics will be evaluated by either $\chi^{2}$ or Fisher extract test for categorical variables and an independent samples t-test for continuous variables. Changes in recovery outcome scores will be compared for each participant as the difference between baseline and each specified time points scores with univariate analyses comparing changes in outcome.

**Methods for additional analyses (e.g. subgroup analyses)**

Each clinical cohort (Cardiac, Vascular and Abdominal surgery) will be assessed in a predefined subgroup.

**Methods in analysis to handle protocol non-adherence and any statistical methods to handle missing data** [20c]

Results will be analysed on an intention-to-treat basis. Secondary analysis will be per protocol.

**Plans to give access to the full protocol, participant level data and statistical code** [31c]

No later than 2 years after the collection of the final 6 month follow up assessments, we will deliver a completely deidentified data set to an appropriate data archive for sharing purposes.

## 11.5 Interim analyses [21b]

The data steering data monitoring committee will conduct an interim analysis 6 monthly intervals to assess feasibility of the trial recruitment and fidelity to the trial protocol.

# 12. Monitoring and Quality Assurance [23]

A trial specific monitoring plan will be established following a risk assessment and full details will be available. The trial will be monitored according to this agreed plan. Local investigators shall ensure that all study data are available for trial related monitoring, audits, and research ethics committee review.

# 13. Study Committees

## 13.1 Project Management Group (PMG) [5d]

The PMG will comprise the chief investigator, trial manager and research fellows responsible for managing the trial. The PMG will be responsible for the set up and day to day running of the trial. It will meet weekly during the planning stages of the study and less frequently when the study is recruiting.

## 13.2 Trial Steering Committee (TSC) [5d]

The TSC includes members of the PMG, experts in the fields of iron deficiency, as well as a lay representative. They will meet at regular intervals, not less than once a year. The TSC, in the development of this protocol and throughout the trial, will take responsibility for:

Major decisions such as a need to change the protocol for any reason Monitoring and supervising the progress of the trial Considering recommendations from the Data Safety and Monitoring Committee (DSMC) informing and advising the PMG on all aspects of the trial

## 13.3 Data Safety and Monitoring Committee (DSMC) [21a]

The DSMC includes three independent members including two medical experts and a statistician. Members of the DSMC cannot be investigators in the study or participate in other committees for this study. The role of the DSMC will be to monitor independently the safety of the study participants and to suggest amendments to this protocol if deemed necessary for reasons of patient safety or feasibility of study procedures. The DSMC members will meet early in the trial to establish a DSMC Charter. The chair of the DSMC reports to the chair of the TSC. To minimise potential bias, DSMC members will not have direct contact with the study site personnel or with participants.

# 14. Administrative Aspects

## 14.1 Ethical Considerations

This study will be conducted according to the Guidance for Good Clinical Practice E6 (R2) and in compliance with applicable laws and regulations. The study will be performed in accordance with the NHMRC Statement on Ethical Conduct in Research Involving Humans 2007 (updated 2018), the Australian Code for the Responsible Conduct of Research, 2018, and the principles laid down by the World Medical Assembly in the Declaration of Helsinki 2013.

## 14.2 Regulatory requirements

The study will be submitted for approval by the local regulatory authorities. SAEs will be notified according to the requirements of the local regulatory authorities.

## 14.3 Protocol amendments [25]

Changes and amendments must be submitted to HREC for review and approval. All Investigators are to conduct the study in accordance with the current protocol except when it is necessary to deviate to protect the safety, rights, and welfare of participant.

## 14.4 Publication policy [31a]

The PMG and TSC will oversee the publication outputs from this work. It is envisaged that the final protocol will be published in a peer reviewed journal and online either through the trial website or via the ANZCTR or Clinicaltrials.gov. The trial outcomes and results from this trial will be published in peer reviewed journals. In line with open access policy, all participants enrolling in the study will be asked if they wish communication of the results.

## 14.5 Archiving

The Investigator will be responsible for the retention of all trial documentation for 15 years or the appropriate retention period as stipulated by local regulations and ICH-GCP. Destruction of essential documents will require authorisation from the Sponsor.

# References

1. Vos T, Flaxman AD, Naghavi M, Lozano R, Michaud C, Ezzati M, et al. Years lived with disability (YLDs) for 1160 sequelae of 289 diseases and injuries 1990-2010: a systematic analysis for the Global Burden of Disease Study 2010. Lancet. 2012;380(9859):2163-96.

2. Muckenthaler MU, Rivella S, Hentze MW, Galy B. A Red Carpet for Iron Metabolism. Cell. 2017;168(3):344-61.

3. Nandhra S, Boylan L, Prentis J, Nesbitt C. The Influence of Preoperative Anemia on Clinical Outcomes After Infrainguinal Bypass Surgery. Ann Vasc Surg. 2020;66:586-94.

4. Bodewes TCF, Pothof AB, Darling JD, Deery SE, Jones DW, Soden PA, et al. Preoperative anemia associated with adverse outcomes after infrainguinal bypass surgery in patients with chronic limb-threatening ischemia. J Vasc Surg. 2017;66(6):1775-85.e2.

5. Velescu A, Clará A, Cladellas M, Peñafiel J, Mateos E, Ibañez S, et al. Anemia Increases Mortality After Open or Endovascular Treatment in Patients with Critical Limb Ischemia: A Retrospective Analysis. Eur J Vasc Endovasc Surg. 2016;51(4):543-9

6. McLean E, Cogswell M, Egli I, Wojdyla D, De Benoist B. Worldwide prevalence of anaemia, WHO vitamin and mineral nutrition information system, 1993–2005. Public health nutrition. 2009;12(4):444-454.
7. Shander A, Knight K, Thurer R, Adamson J, Spence R. Prevalence and outcomes of anemia in surgery: a systematic review of the literature. The American journal of medicine. 2004;116(7):58-69.

8. Nutritional anaemias: report of a WHO scientific group [meeting held in Geneva from 13 to 17 March 1967]. World Health Organization. 1968.
9. Dunne JR, Malone D, Tracy JK, Gannon C, Napolitano LM. Perioperative anemia: an independent risk factor for infection, mortality, and resource utilization in surgery. Journal of Surgical Research. 2002;102(2):237-244.

10. Halm EA, Wang JJ, Boockvar K, Penrod J, Silberzweig SB, Magaziner J, et al. The effect of perioperative anemia on clinical and functional outcomes in patients with hip fracture. Journal of orthopaedic trauma. 2004;18(6):369.
11. Beattie WS, Karkouti K, Wijeysundera DN, Tait G. Risk associated with preoperative anemia in noncardiac SurgeryA single-center cohort study. Anesthesiology: The Journal of the American Society of Anesthesiologists. 2009;110(3):574-581.

12. Carson JL, Duff A, Poses RM, Berlin JA, Spence RK, Trout R, et al. Effect of anaemia and cardiovascular disease on surgical mortality and morbidity. The Lancet. 1996;348(9034):1055- 1060.
13. Engoren M, Habib RH, Hadaway J, Zacharias A, Schwann TA, Riordan CJ, et al. The effect on long-term survival of erythrocyte transfusion given for cardiac valve operations. The Annals of thoracic surgery. 2009;88(1):95-100. e3.

14. Karkouti K, Wijeysundera DN, Beattie WS. Risk associated with preoperative anemia in cardiac surgery. Circulation. 2008;117(4):478-84.
15. Gombotz H, Rehak PH, Shander A, Hofmann A. Blood use in elective surgery: the Austrian benchmark study. Transfusion. 2007;47(8):1468-1480.
16. Keating EM, Meding JB, Faris PM, Ritter MA. Predictors of transfusion risk in elective knee surgery. Clinical orthopaedics and related research. 1998 (357):50-59.
17. van Klei WA, Moons KG, Rheineck Leyssius A, Knape JT, Rutten CL, Grobbee DE. A reduction in type and screen: preoperative prediction of RBC transfusions in surgery procedures with intermediate transfusion risks. British journal of anaesthesia. 2001;87(2):250-257.
18. Ong A, Sim K, Boey S. Preoperative prediction of intra and postoperative red blood cell transfusion in surgical patients. Annals of the Academy of Medicine, Singapore. 1997;26(4):430- 434.
19. Hébert PC, Wells G, Blajchman MA, Marshall J, Martin C, Pagliarello G, et al. A multicenter, randomized, controlled clinical trial of transfusion requirements in critical care. New England Journal of Medicine. 1999;340(6):409-417.
20. Sharma S, Sharma P, Tyler LN. Transfusion of blood and blood products: indications and complications. American family physician. 2011;83(6):719-724.

21. Kotzé A, Harris A, Baker C, Iqbal T, Lavies N, Richards T, et al. British Committee for Standards in Haematology Guidelines on the Identification and Management of Pre-Operative Anaemia. Br J Haematol. 2015;171(3):322-31.

22. Muñoz M, Acheson AG, Auerbach M, Besser M, Habler O, Kehlet H, et al. International consensus statement on the peri-operative management of anaemia and iron deficiency. Anaesthesia. 2017;72(2):233-47.

23. Clevenger B, Gurusamy K, Klein AA, Murphy GJ, Anker SD, Richards T. Systematic review and meta-analysis of iron therapy in anaemic adults without chronic kidney disease: updated and abridged Cochrane review. Eur J Heart Fail. 2016;18(7):774-85.

24. Nandhra S, Chau M, Klein A, Yeates J, Collier T, Evans C, et al. Preoperative anaemia management in patients undergoing vascular surgery. The British journal of surgery. 2020;107.

25. Klein AA, Chau M, Yeates JA, Collier T, Evans C, Agarwal S, et al. Preoperative intravenous iron before cardiac surgery: a prospective multicentre feasibility study. Br J Anaesth. 2020;124(3):243-50.

26. Richards T, Baikady RR, Clevenger B, Butcher A, Abeysiri S, Chau M, et al. Preoperative intravenous iron to treat anaemia before major abdominal surgery (PREVENTT): a randomised, double-blind, controlled trial. The Lancet. 2020;396(10259):1353-61.

27. Postoperative variations in anaemia treatment and transfusions (POSTVenTT): protocol for a prospective multicentre observational cohort study of anaemia after major abdominal surgery. Colorectal Dis. 2022;24(2):228-34.

28. Litton E, Baker S, Erber W, Farmer S, Ferrier J, French C, et al. Hepcidin predicts response to IV iron therapy in patients admitted to the intensive care unit: a nested cohort study. Journal of Intensive Care. 2018;6(1):60.

29. Martin-Cabrera P, Hung M, Ortmann E, Richards T, Ghosh M, Bottrill F, et al. Clinical use of low haemoglobin density, transferrin saturation, bone marrow morphology, Perl's stain and other plasma markers in the identification of treatable anaemia presenting for cardiac surgery in a prospective cohort study. J Clin Pathol. 2015;68(11):923-30.

30. Litton E, Baker S, Erber WN, Farmer S, Ferrier J, French C, et al. Intravenous iron or placebo for anaemia in intensive care: the IRONMAN multicentre randomized blinded trial : A randomized trial of IV iron in critical illness. Intensive Care Med. 2016;42(11):1715-22.

31. Shah A, Chester-Jones M, Dutton SJ, Marian IR, Barber VS, Griffith DM, et al. Intravenous iron to treat anaemia following critical care: a multicentre feasibility randomised trial. Br J Anaesth. 2022;128(2)

|  |  | **Approached patients (n = 137)** |
| --- | --- | --- |
| Ineligible | Non-anaemic | 52 (38%) |
|  | Received Iron | 2 (1%) |
| Declined | Not interested | 26 (19%) |
|  | Transport issues | 20 (15%) |
|  | Schedule issues | 14 (10%) |
|  | Personal medical concerns | 12 (9%) |
|  | Ongoing treatment | 7 (5%) |
| Randomised | Lost to follow up | 3 (2%) |
|  | Completed study | 1 (1%) |

**Table 1. Hurdles to trial recruitment**. The above table details the distributions of trial eligibility, reasons for declined participation and the outcome of those consented and randomised to the trial. Number of responders are reported out of those patients approached for recruitment into the trial (n = 137).

|  | **Outcome** | **Week 4** | **Week 8** | **Week 12** | **Week 16** |
| --- | --- | --- | --- | --- | --- |
| **SF-36 Domains** | Physical Function | 5.0 (±15.8) | 40.0 (±31.6) | 65.0 (±33.7) | 50.0 (±33.3) |
|  | Role-Physical | 25.0 (±0.0) | 37.5 (±14.4) | 43.8 (±12.5) | 50.0 (±0.0) |
|  | Role Emotional | 41.7 (±52.0) | 50.0 (±0.0) | 83.3 (±14.4) | 58.3 (±14.4) |
|  | Energy | 20.0 (±28.3) | 40.0 (±36.5) | 35.0 (±30.0) | 40.0 (±28.3) |
|  | Emotional Wellbeing | 44.0 (±21.9) | 60.0 (±28.3) | 60.0 (±24.5) | 56.0 (±16.7) |
|  | Social Function | 12.5 (±17.7) | 37.5 (±17.7) | 50.0 (±0.0) | 62.5 (±17.7) |
|  | Pain | 45.0 (±7.1) | 45.0 (±7.1) | 45.0 (±7.1) | 55.0 (±7.1) |
|  | General Health | 45.0 (±27.4) | 50.0 (±17.7) | 75.0 (±17.7) | 55.0 (±11.2) |
| **SF-36 T-Score** | PCS | 27.4 | 33.4 | 38.1 | 38.6 |
|  | MCS | 27.6 | 32.9 | 40.1 | 34.7 |
| **EQ-5D-5L** | Index | 0.848 | 0.956 | 0.848 | 0.92 |
|  | EQ-VAS | 80 | 80 | 80 | 95 |
| **Physical Function** | Maximum Grip (kg) | 38 | 34.7 | 33.7 | 34.2 |
|  | TUG (seconds) | - | 13.64 | 12.42 | 10.78 |
|  | SMWT (m) | - | 310 | 387 | 410 |
|  | Sit to Stand | - | 8 | 9 | 11 |
|  | CFS | 4 | 2 | 1 | 1 |

**Table 2. Quality of life and physical assessments**. The above table demonstrates the scores for each section of the quality of life questionnaires for the patient from baseline (week 4) to week 12 post-discharge. SF-36 domains are reported as the mean ± SD for each domain score and scored out of 100, with the latter being the best possible score[11]. PCS and MCS are reported as T-scores, derived from population norms and factor coefficient weights obtained from data describing the Australian population derived value set[12]. The EQ-5D-5L is reported as visual analogue score (EQ-VAS), where 100 is the best imaginable health and 0 the worst, and reported as an index score that is weighted according to an Australian population derived value set, where 1 is valued as full health and 0 is valued as death[12]. Physical function outcomes are reported continuously (TUG = time to up and go, SMWT = six-minute walk test, CFS = clinical frailty scale), with the exception of CFS which is reported as a score, where 1 is the best outcome and 9 is the worst.

**Supplementary Figure 1. Longitudinal haematological response.**

**Supplementary Figure 2. SF-36 T-score report**. The SF-36 is reported in 2 components: the physical component score (PCS) and mental component score (PCS). The T-scores for each component are derived from population norms and factor score coefficient weights obtained from data describing the Australian population reported in 2007[30].
